# Supplementary material for: Causal interplay between lactose intolerance and gut microbiota: a combined bidirectional Mendelian randomization and in vivo validation study
Source: Front Nutr. 2026 Jun 1;13:1803337. doi: 10.3389/fnut.2026.1803337 (PMC13265576; doi:10.3389/fnut.2026.1803337)
Supplement: Supplementary file 1 [file Data_Sheet_1.zip › supplementary materials/Forward/leave one out cross validation/ebi-a-GCST90027557.finngen_R12_E4_LACTONAS.pdf]

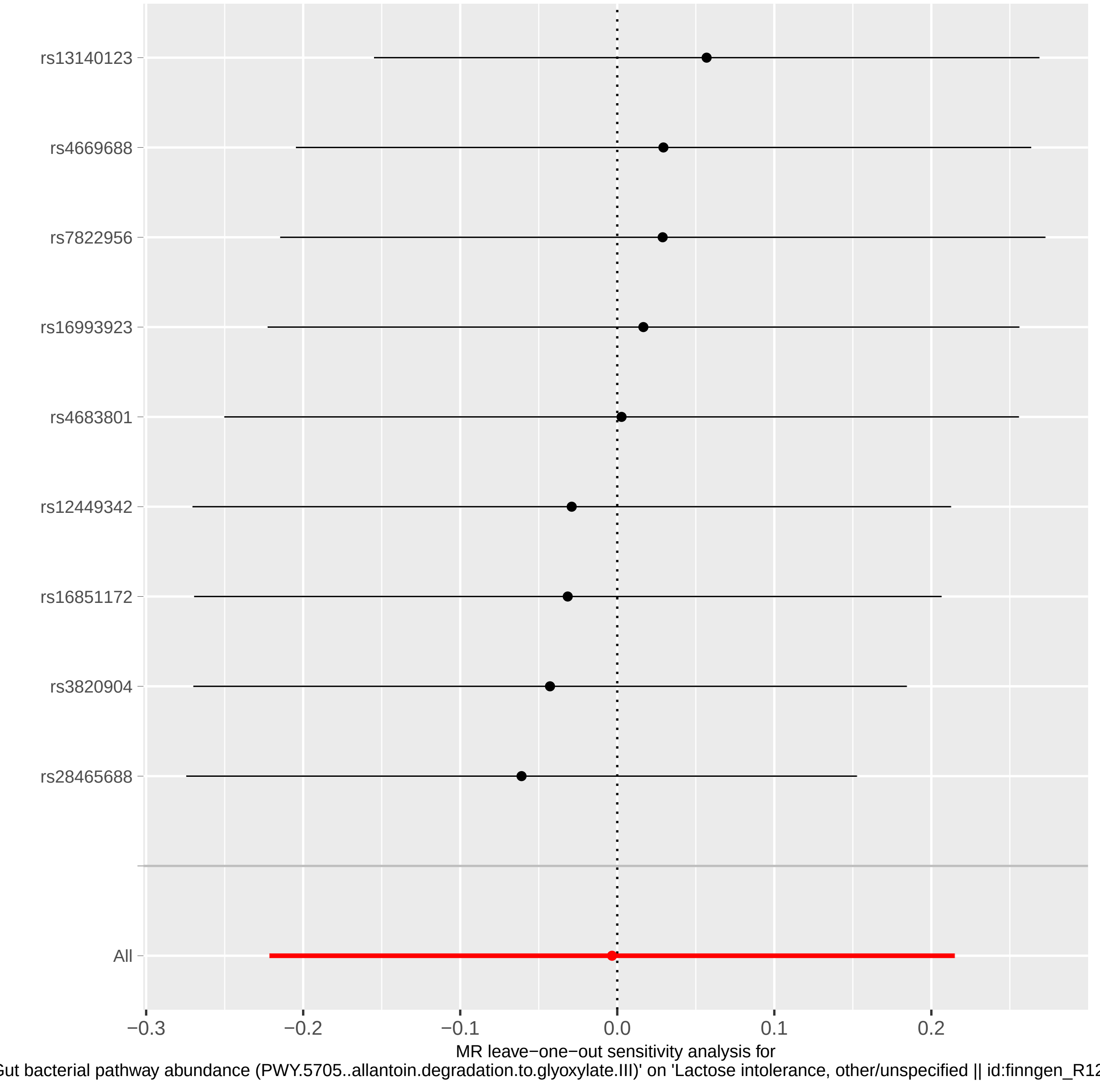
rs13140123
rs4669688
rs7822956
rs16993923
rs4683801
rs12449342
rs16851172
rs3820904
rs28465688
All
−0.3
−0.2
−0.1
0.0
0.1
0.2
MR leave−one−out sensitivity analysis for
Gut bacterial pathway abundance (PWY.5705..allantoin.degradation.to.glyoxylate.III)' on 'Lactose intolerance, other/unspecified || id:finngen_R12
